# Promoting early autism detection and intervention in underserved communities: study protocol for a pragmatic trial using a stepped-wedge design

**DOI:** 10.1186/s12888-019-2150-3

**Published:** 2019-06-07

**Authors:** Lisa V. Ibañez, Ann Vander Stoep, Kathleen Myers, Chuan Zhou, Shannon Dorsey, Kyle J. Steinman, Wendy L. Stone

**Affiliations:** 1 Department of Psychology, University of Washington, CHDD, Box 357920, Seattle, WA 98195 USA; 2 Department of Psychiatry and Behavioral Sciences, Department of Epidemiology, University of Washington, Seattle, WA 98195 USA; 3 Department of Psychiatry and Behavioral Sciences, University of Washington, Seattle, WA 98195 USA; 4 Department of Pediatrics, School of Medicine, University of Washington, Seattle, WA 98195 USA; 5 Departments of Neurology, Psychiatry & Behavioral Sciences, and Pediatrics, University of Washington, Seattle, WA 98195 USA; 6 Center for Integrative Brain Research, Seattle Children’s Research Institute, Seattle, WA 98101 USA; 7 Center for Child Health, Behavior and Development, Seattle Children’s Research Institute, Seattle, WA 98145 USA

**Keywords:** Autism Spectrum Disorder, Early detection, Preventive intervention, Universal screening, Stage 2 screening, Pragmatic trial, Stepped-wedge design

## Abstract

**Background:**

Despite the known benefits of early, specialized intervention for toddlers with Autism Spectrum Disorder (ASD), access to such intervention remains limited. This pragmatic trial examines a novel healthcare delivery model (Screen-Refer-Treat [SRT]), which capitalizes upon existing health care and early intervention (EI) infrastructure to increase community capacity for ASD detection and treatment before age 3, when it is likely to have the greatest impact. This model comprises three components: (1) universal use of Stage 1 ASD screening by primary care providers (PCPs) at 18-month well-child visits (i.e., Screen); (2) immediate referral of positive screens to a community-based EI program (i.e., Refer); and (3) provision of an inexpensive, evidence-based ASD-specialized treatment by EI providers, after verifying ASD risk with a Stage 2 screen (i.e., Treat). This paper describes our research design and the initial successes, challenges, and adaptations made during the early implementation phase.

**Method/design:**

A stepped-wedge cluster RCT was used to implement the SRT model sequentially in four diverse Washington State counties (“clusters”). Counties are randomly assigned to the time of receipt of the SRT intervention, which comprises training workshops and technical assistance focused on the use of evidence-based ASD screening and intervention tools. Separate cohorts of families with toddlers (16–35 months old) with and without ASD concerns are recruited before and after the SRT intervention from participating PCP practices and EI programs. PCPs and EI providers complete measures on their screening, referral, and intervention practices before and after the SRT intervention. Each family cohort completes surveys about their well-being, parenting efficacy, health care satisfaction, and toddler’s social-communicative behaviors.

**Conclusion:**

This trial is the first of its kind to work simultaneously with two service delivery systems with the goal of improving early detection and treatment for ASD. Our approach was successful in attaining buy-in from PCPs and EI providers, building and maintaining partnerships with providers, and achieving high levels of retention and survey completion. Fostering provider engagement and problem-solving issues together as partners were integral to overcoming the main challenges. Numerous lessons have been learned thus far, which have applicability for implementation researchers in ASD and those in other fields.

**Trial registration:**

The registration number for this trial is NCT02409303 and it was posted on ClinicalTrials.gov on April 6, 2015.

## Background

Autism Spectrum Disorder (ASD) is a complex neurodevelopmental disorder characterized by severe impairment in two broad domains: social communication and behavioral rigidity [1]. The prevalence of ASD has increased steadily over the years and is now estimated to affect 1 in 59 children in the United States (U.S.), with children from Hispanic backgrounds identified at lower rates and later ages due to inadequate detection [2]. Although there is currently no known cure for ASD, early identification and specialized intervention have led to significant improvements in social, language, cognitive, and behavioral functioning [3–7]. To promote early detection and optimize child outcomes, the American Academy of Pediatrics (AAP) published ASD practice guidelines that include conducting universal ASD-specific screening with a standardized tool at 18 and 24 months of age [8]. However, guideline compliance is low [9], with primary care providers (PCPs) reporting numerous barriers to screening, including insufficient time during well-child visits, work flow challenges, and reimbursement issues [10–13].

The prevalent model for ASD care requires a formal diagnosis from a qualified professional to obtain access to ASD-specialized intervention. Most communities have insufficient numbers of professionals with expertise in diagnosing or treating ASD; thus, many toddlers with ASD fail to receive appropriately specialized intervention during the birth-to-three years, when its impact might be greatest [14, 15]. Concerned parents may encounter delays of up to 2 years before receiving an ASD diagnosis and/or specialized services [16–18]. This service vacuum not only creates stress for concerned parents, but also provides a disincentive for PCPs to conduct early ASD screening [9, 16, 19]. In this paper, we describe an alternative service delivery model and a pragmatic trial we are conducting to evaluate the model.

### Designing a new service delivery model for ASD: the screen-refer-treat model

**Fig. 1:**
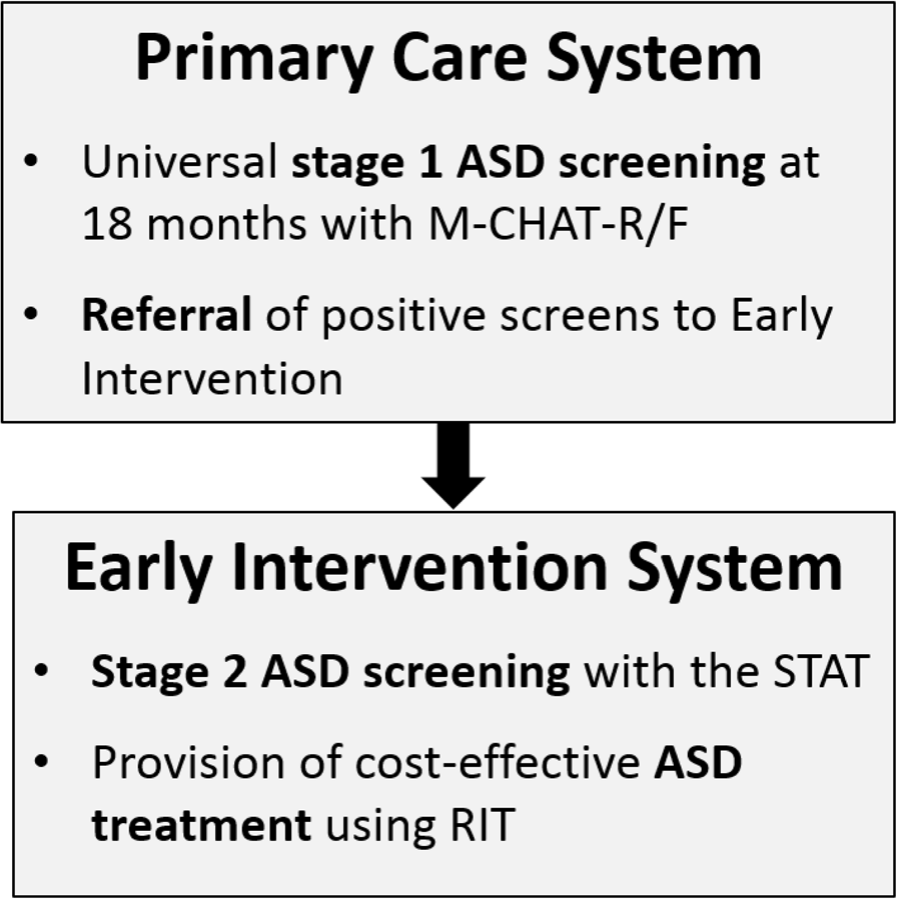
SRT flow across systems

The Screen-Refer-Treat (SRT) model employs state-of-the-art developments in ASD detection and intervention and capitalizes on a readily accessible service delivery infrastructure [20]. The goal of the SRT model is to use a preventive intervention approach and mitigate emerging ASD behaviors during a critical period of development. This model introduces a significant shift in the conceptualization and implementation of ASD services by: (1) offering an ASD-specialized intervention to toddlers when ASD is first *suspected*, rather than waiting until a formal ASD diagnosis is conferred; (2) involving *two integral and interrelated parts of the service delivery system—*primary care and early intervention (EI)—to increase care coordination (See Fig. 1); and (3) addressing obstacles associated with early detection and intervention through the *use of technology* to expedite screening. Two stages of screening are used: Stage 1 (designed for primary care settings), and Stage 2 (designed for referral settings) [21].

The SRT model comprises three components: PCPs conduct universal Stage 1 ASD screening at the 18-month well-child visit (Screen); PCPs immediately refer toddlers who screen positive to a community-based EI program (Refer); and EI providers initiate ASD treatment (Treat) after verifying ASD risk with a Stage 2 screen.

#### Screen

PCPs screen children at 18-month well-child checks using the Modified Checklist for Autism in Toddlers-Revised, with Follow Up (M-CHAT-R/F) [22, 23]. The M-CHAT-R/F is a well-validated Stage 1 parent-report screening tool for detecting ASD in population-based settings. It comprises two components: a 20-item behavior checklist completed by parents; and follow-up questions conducted as an interview if the child screens positive on the checklist. In typical clinical practice, however, time constraints often prevent PCPs from administering the follow-up interview questions [9, 10], which can lead to elevated rates of false positive results [22, 24]. To circumvent this challenge, we developed a web-based version of the M-CHAT-R/F [25, 26] that automatically triggers the appropriate follow-up questions for parents to complete if the checklist score indicates that the child is “at risk”. Scoring is automated, and results are delivered electronically to the PCP.

#### Refer

PCPs immediately refer toddlers with a positive M-CHAT-R/F to a community-based EI program. These programs are federally-funded under the Individuals with Disabilities Education Act (IDEA) [27] to serve infants and toddlers under 36 months old who have delays or disabilities, and are available in all communities at no cost to families.

#### Treat

EI providers assess the need for ASD-specialized treatment using the Screening Tool for Autism in Toddlers (STAT) [28–30], a 12-item, interactive Stage 2 screen that is completed in 20 min and scored in real time. For children screening positive on the STAT, EI providers deliver an evidence-based, ASD-specialized behavioral intervention, Reciprocal Imitation Training (RIT) [31, 32]. RIT uses a naturalistic behavioral approach and a play-based context to teach imitation skills, which are a core deficit area for young children with ASD. Because imitation is a pivotal skill, RIT has also led to concurrent improvements in broader social-communication skills [31, 32].

### Study aims

This pragmatic trial was designed to test the effectiveness of the SRT model in improving both system-level and family-level outcomes. At the *system level*, our aims are to: (1) increase the number of toddlers receiving ASD screening at their 18-month well-child visit; (2) increase the number of toddlers with positive screens who receive evidence-based behavioral intervention prior to age 3; (3) reduce the length of time between parents’ expressed concerns about ASD and the child’s receipt of ASD-specialized intervention; and (4) reduce disparities in identification of ASD for Hispanic families, given the magnitude of the delays they experience relative to other underrepresented populations [2]. At the *family level*, the SRT project aims to: (1) improve parents’ well-being, parenting efficacy, parenting stress, and health care satisfaction; and (2) improve children’s social-communication skills. This paper describes how the SRT trial was designed and is being conducted, highlighting successes and challenges we have encountered during trial implementation.

## Method

### Study design

The SRT service delivery model was initiated in four underserved counties in geographically distinct areas of Washington (WA) State, using a pragmatic trial framework and a stepped-wedge cluster randomized controlled design (RCT) [33]. The research hub was centralized at the University of Washington, where IRB approval was obtained prior to the commencement of study activities and for all protocol amendments. This paper reports on research activities and data collection that occurred during the first 22 months of this project, prior to implementation of SRT training activities. During this “Pre-SRT” period, two baseline Pre-SRT surveys have been collected from providers, and enrollment of the Pre-SRT cohort of families has been completed.

#### Pragmatic trial

In contrast to explanatory trials, which test how an intervention works under optimal conditions, pragmatic trials are designed to evaluate the effectiveness of an intervention in routine practice conditions [34]. Consistent with the pragmatic approach [35], SRT recruits community providers and the families whom they serve. All individual components of the SRT intervention have been established as efficacious and ethical and they are organized around usual clinic practice, allowing for flexibility in procedures, methods, and staffing. Pragmatic components of the SRT trial are summarized in Table 1.

**Table 1:** Pragmatic Features Employed in SRT Clinical Trial

Feature ^a^	Description
Eligibility	• Providers: Community-based PCPs and EI providers in 4 selected counties
	• Parents: Parents of children 16–36 months old receiving care from an enrolled provider; meet criteria for ASD concerns or No concerns based on study screening interview; English- or Spanish-Speaking; No significant comorbid medical conditions.
Recruitment	• Providers: Recruited from usual care settings; Compensation provided only for research-related activities (e.g., mailing recruitment flyers; completing surveys)
	• Parents: Recruited by participating PCPs and EI providers via: posting flyers in waiting area, handing flyers to parents, and/or sending flyers to all families with age-eligible children (i.e., not otherwise targeted). Compensation provided for completing study surveys.
Setting	• Conducted exclusively within usual care settings
Organization	• Care delivery is consistent with that provided in PCP (i.e., screening; referral) and EI (i.e., assessment; intervention) settings.
	• Study provides training and technical assistance in using evidence-based tools.
	• Study provides compensation to programs for EI providers’ workshop attendance.
	Additional resources provided through the study:
	• PCPs & EIs: Materials about early features of ASD and communicating with parents about ASD concerns.
	• PCPs only: Information about local ASD resources; Hand-held tablets parents use to complete the online M-CHAT-R/F; Access to a REDCap database for obtaining M-CHAT-R/F results; CME and/or MOC credits (at no cost) for workshop and project participation
	• EIs only: Interview probes for eliciting behavioral reports from parents; Telemedicine equipment for receiving online consultation. and feedback; STAT materials and certification at no cost; (4) optional CEU credits for workshops (self-pay).
Flexibility/Delivery	• PCPs & EIs: Freedom to use additional screening, assessment, and/or intervention tools; freedom to use the M-CHAT-R/F, STAT, and/or RIT with non-enrolled families and/or children outside the study age range; freedom to develop their own workflow plans.
	Study-specific expectations:
	• PCPs: Use of the web-based M-CHAT-R/F universally at 18 months; Referral of positive screens to EI programs. Both are consistent with AAP practice guidelines.
	• EIs: Use of the STAT for children referred from PCPs with positive M-CHAT-R/F screens; Use of RIT for children who continue to screen positive for ASD.
Flexibility/Adherence	• Adherence to the intervention protocol (i.e., use of the M-CHAT-R/F, STAT, or RIT) is not required for continued study participation.
	• PCPs & EIs: Adherence is monitored through self-report surveys at predetermined intervals.
	• PCPs only: Use of web-based M-CHAT-R/F is monitored at the practice level through the REDCap database. Office managers are contacted if M-CHAT-R/F use is low or declines, to identify possible technical assistance needs.
Follow-up	• PCPs & EIs: Completion of self-report surveys 3 times over an 18-month period after the training workshops.
	• PCPs only: Monitoring of M-CHAT-R/F use through REDCap database records for 18 months following the training workshop.
Primary outcomes	• Providers: Feasibility, acceptability, and use of the M-CHAT-R/F, STAT, and RIT.
	• Parents: Improvements in overall well-being, health care satisfaction, parenting stress, and parenting efficacy for ASD concerns group.
	• Children: Improved social communication skills and earlier receipt of specialized intervention for children with ASD concerns.
Primary analysis	All data are analyzed using an intent-to-treat model.

^a^ These features are outlined in Loudon et al. [35]

**Table 2:** Community Demographics

	Spokane	Yakima	Skagit	Lewis
Distance from diagnostic services in Seattle (miles)	279	143	61	84
Population #	475,735	246,977	118,222	75,621
Population density (#/square mile)	3481	56.4	60.9	31
% with Bachelor’s degree or higher	29%	16%	24%	15%
% infants served by WIC	51%	76%	51%	57%
% White	86%	46%	76%	85%
% Hispanic	5%	46%	17%	9%
% Other (combined)	9%	8%	7%	6%

#### Stepped-wedge cluster RCT design

**Fig. 2:**
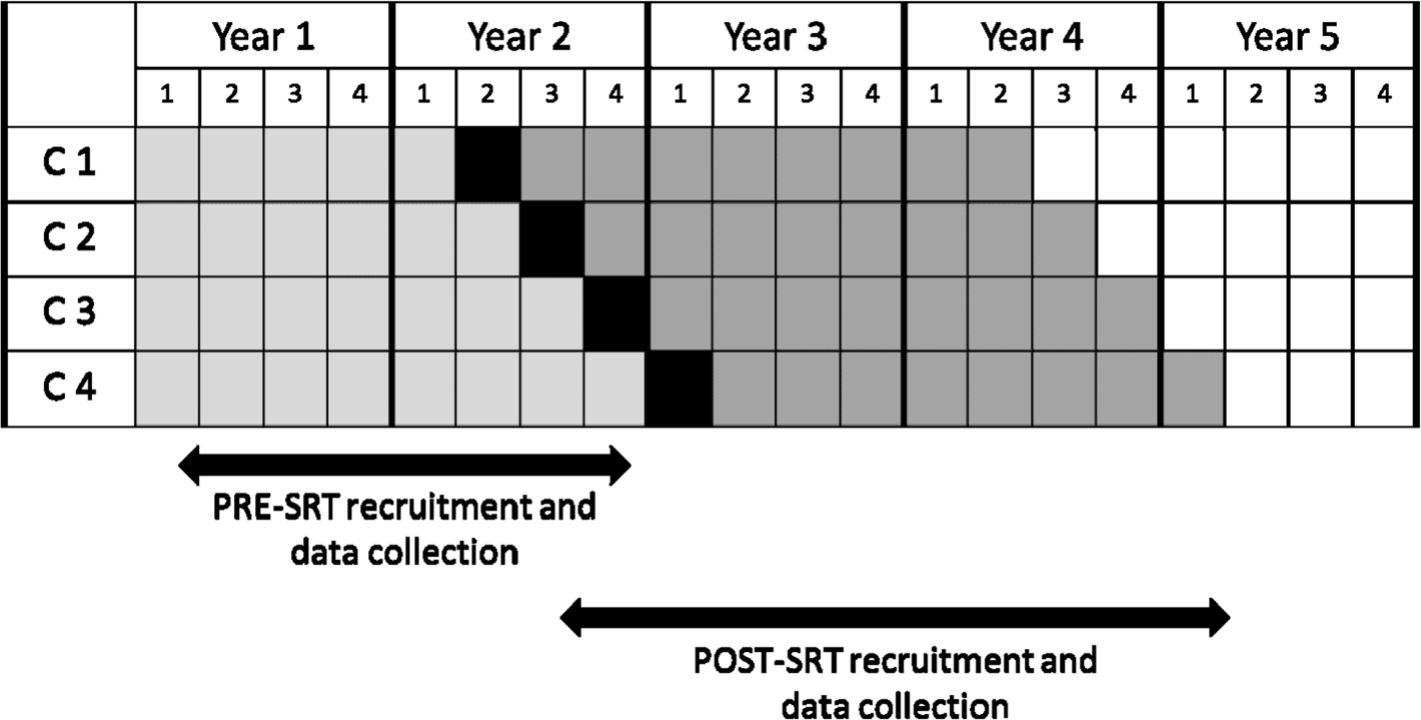
Stepped-Wedge Cluster RCT Study Design. The four counties (C1-C4) are randomized to the timing of their SRT training workshops. Lighter shading indicates the period of Pre-SRT family recruitment and data collection, darker shading indicates the Post-SRT family recruitment and data collection window, and black shading indicates the 3-month training and technical assistance (TA) period for providers

After a period of baseline data collection in Year 1, each county (“cluster”) was randomly assigned (using random permutation/shuffling by the biostatistician) to one of four time periods during Year 2 when providers received their training in the SRT components (see Fig. 2). Our decision to employ the stepped-wedge design was based on several considerations. First, early feedback from providers revealed that their motivation to participate over a 5-year study period was contingent on their receipt of the SRT training. Second, this approach removes some of the logistical constraints associated with implementing a complex multi-level healthcare system intervention in multiple locations, by allowing us to stagger training activities for each county. Third, this design allows for concurrent comparisons between counties exposed to the SRT model and those not yet exposed; a simple pre-post comparison may be confounded by policy or system changes occurring between the pre-intervention and post-implementation periods. Randomization was at the county level (cluster) rather than at the organizational level within each county to minimize cross-contamination (i.e., providers already trained in one organization exposing the yet-to-be trained providers in another organization to the SRT components).

Changes in PCP and EI provider practices are examined using a *within-subjects* approach, by comparing service delivery practices before and after SRT intervention. Parent experiences are examined using a *between-subjects* approach, by comparing a sample of parents recruited prior to SRT intervention (Pre-SRT cohort) to a separate sample of parents recruited after SRT intervention (Post-SRT cohort); parents were not aware of the study design and, as such, did not know what cohort they were in.

### Participant recruitment

Recruitment entailed first identifying interested counties, then identifying PCP practices and EI programs with interested providers, and finally recruiting parent/toddler dyads from the caseloads of the participating PCP practices and EI programs.

#### County selection

Counties were selected for participation based on: (1) demographic diversity; (2) the presence of local champions who facilitated connections between the research team and local providers; and (3) interest from both PCP practices and EI programs. The counties vary on sociodemographic factors (see Table 2).

#### Recruitment and consenting of providers

The research team visited interested PCP practices and EI programs to provide study details and obtain feedback regarding study procedures. Suggestions from providers resulted in adaptations to workshop content, study procedures, survey instruments, and study incentives. Our plan was to partner with 2 PCP practices and 2 EI agencies in each county and enroll a total of 40 PCPs and 80 EI across all 4 counties. PCPs and EI providers were given study consent forms and provided with the opportunity to review them at their leisure, and contact the research team with any questions. PCPs and EI providers who enrolled received compensation for completing surveys at designated intervals ($20/time period).

#### Recruitment, eligibility, and consenting of families

In both the Pre-SRT and Post-SRT implementation periods, two parent-toddler samples are recruited: an “ASD Concerns” sample and a “No Concerns” sample. The ASD Concerns sample, recruited from both PCP and EI settings, comprises toddlers who either have an ASD diagnosis or meet one or more of the following criteria indicating ASD “risk”: (1) behaviorally-based parental concerns about the presence of ASD; (2) behaviorally-based provider concerns (PCP or EI) about the presence of ASD; (3) having an older sibling with an ASD diagnosis; or (4) parental report of a prior positive screen on a validated ASD screening tool. The planned sample size for the ASD Concerns group was 490 families total (245 Pre-SRT and 245 Post-SRT). The No Concerns sample comprises toddlers whose parents do not endorse any ASD or other concerns; this sample is recruited exclusively from PCP practices to characterize health care experiences of families without concerns about their child’s development. The planned sample size for the ASD Concerns group was 70 families total (35 Pre-SRT and 35 Post-SRT. English- and Spanish-speaking families are recruited from the participating PCP practices and EI programs using materials available in both languages.

For PCP practices, families of 16- to 20-month-olds attending the child’s 18-month well-child visit receive a study flyer and a “permission-to-contact” form by the PCP or medical assistant. For EI programs, recruitment occurs in several ways: flyers are posted in waiting areas, recruitment letters are sent to all parents of toddlers in the program within the desired age range (16–35 months), and EI providers give the study flyer and permission-to-contact form directly to families with ASD concerns. Once parents directly reached out or providers sent the completed permission-to-contact forms, the study personnel conduct a structured telephone screening interview to determine study eligibility and group assignment.

Parents who were interested and eligible to participate were either mailed or emailed copies of the study consent materials. They were provided the opportunity to review the forms at their leisure, and were instructed to call the research team with any questions or concerns. For parents with literacy issues, research staff members guided them through the consent information and provided them the opportunity to verbally ask questions and consent to the study over the phone. Similar to providers, parents who enrolled received $20 compensation for completing surveys at designated intervals.

### Study procedures

#### Measurement and data collection

Self-report survey data are collected from PCPs, EI providers, and parents. REDCap [26] was used to create a web-based, longitudinal database that is HIPAA compliant and allows for online survey completion. Parent and provider measures are described in Table 3.

**Table 3:** Description of Measures Collected

Measure	Brief Description
Completed by PCP Practices	
PCP Practices Survey [53]	Measures PCPs’ screening and referral practices and their understanding of early ASD characteristics.
PCP Checklist [54]	Describes the screening procedures used, referrals made, and the presence of ASD concerns/risk factors at the child’s 18-month well-child visit.
Implementation Survey *(Adapted from Dingfelder & Mandell)* [55]^,a^	Measures the barriers and facilitators to using the web-based M-CHAT-R/F, as well as its feasibility and acceptability.
Completed by EI Programs	
EI Provider Practices Survey [56]	Measures providers’ screening and treatment practices and their understanding of early ASD characteristics.
EI Checklist [57]	Describes the assessments and interventions each child enrolled from EI has received.
Implementation Survey *(Adapted from Dingfelder)* [58]^,a^	Measures the barriers and facilitators to using the STAT, caregiver interview, and RIT, as well as their feasibility and acceptability.
Completed by Parents	
Family Demographic Form	Measures family characteristics (e.g., parent education, number of children)
Child Health Services Survey [59]	Assesses the child’s development and their history of screening, referrals, diagnosis, and intervention services.
Intervention Services Survey [60]	Measures the types of directed intervention and parent-mediated intervention toddlers are receiving.
Parenting Stress Index - Short Form [61]	Measures 3 different types of parenting-related stress.
Parenting Efficacy Scale [62]	Measures parents’ perceived efficacy in several domains of child care.
Parent Interview for Autism–Clinical Version [63]	Measures toddlers’ ASD symptom severity in 4 social-communicative domains.
Measure of Processes of Care *(Adapted from Bjerre* et al.*)* [64]	Measures 5 dimensions of healthcare professionals’ behavior. Completed for their PCP practice and EI program (if applicable).
WHO Quality Of Life-BREF *(WHO, 1997)* [65]	Measures satisfaction in the domains of physical health, psychological health, social relationships, and environment.

Note. ^a^ Measures to be collected only during the Post-SRT intervention phase

##### Parent measures.

Parent measures assess parents’ well-being and health care satisfaction and toddlers’ social communication and receipt of community-based services. All measures are available in English and Spanish and are collected every three months until the toddler reaches 36 months of age. Identical procedures are used for Pre-SRT and Post-SRT cohorts.

##### PCP and EI provider measures.

Three types of provider measures are used to detect changes in knowledge, beliefs, and practices related to early identification and intervention for toddlers with ASD. The lack of standardized measures of the specific concepts of interest to this project made it necessary to develop our own tools [36]. Provider Practices Surveys contain questions about early ASD screening, referral, treatment, and are completed at five measurement points, twice before introduction of the SRT model (i.e., at the initial data collection point and one-month prior to their training workshop) and three times after its introduction (i.e., 6, 12, and 18 months after SRT training). Provider Checklists identify provider practices employed at the level of individual patients/toddlers. The PCP Checklist is completed immediately after all 18-month well-child visits, regardless of study participation, to gather anonymized information about the presence of ASD risk factors, whether the child was screened, the screening results, and referrals made. The EI Checklist is completed for all children enrolled in the study who attend the EI program, to indicate the type of services provided. Provider Implementation Surveys assess the feasibility and acceptability of using the M-CHAT-R/F, STAT, and RIT, and are completed at 6-, 12-, and 18-months after the SRT training.

##### Training providers in SRT components.

Participating PCPs, EI providers, and clinic support staff in each county receive training and technical assistance (TA) in implementing the SRT model over a 3-month period (Fig. 2). Training is provided in-person through interactive workshops conducted at each practice site.

##### Screen and referral training for PCPs.

Training for PCPs comprises two components. The first is a 2-h onsite workshop for each practice. Training emphasizes the rationale and processes for recognizing the early behavioral features of ASD, conducting universal screening at 18 months, using the web-based M-CHAT-R/F, and discussing positive screens and appropriate referrals with parents. Tablets and WiFi “hotspots” are provided to each practice. The second component involves an onsite TA visit to each practice 2 weeks later, to train office and medical staff on using the web-based M-CHAT-R/F system and developing an optimal work flow plan to accommodate the new procedures. Work flow plans delineate: (1) who is responsible for handing parents the tablet containing the web-based M-CHAT-R/F, and collecting it when completed, (2) when and where the tablet will be given to parents (e.g., waiting area vs. exam room), (3) how the M-CHAT-R/F results are displayed on the final screen, and (4) how the results are conveyed to the PCP. The research team remains “on-call” for TA requests and works with office staff to monitor progress and detect barriers to implementation.

##### Assessment and treatment training for EI providers.

Training for EI providers occurs through two one-day workshops, both involving “hands-on” practice with children. The STAT workshop provides training on the STAT and a developmentally-targeted parent interview to identify ASD risk. Information about discussing ASD with parents is also included. The RIT workshop provides training on using RIT with toddlers at ASD risk, as well as coaching parents in the use of RIT. WiFi hot spots and video recording equipment are provided to each program to enable them to receive performance feedback and online coaching for the STAT and RIT.

#### Data management, storage, and monitoring

For both the Online M-CHAT-R/F and online versions of the provider and caregiver surveys, the REDCap system was selected as the web application because of its: (1) user friendly interface; (2) capability for programming complex branching logic; (3) security assurances (e.g., HIPAA compliant); (4) cost-effectiveness (i.e., free to partnering universities and institutions); and (5) opportunity for ongoing guidance and support from experts at the UW Institute for Translational Health Sciences. To facilitate data collection, the research team designed and programmed a web-based, longitudinal database to allow PCPs, EI providers, and parents to complete all surveys online; paper-based surveys were also available for request. Data verification for paper surveys was carried out through double data entry and the reconciliation process, which detected random mistakes and also identify systematic deviations from the correct entry of certain fields/items/scores. At the time of enrollment, providers and parents were each assigned a unique numerical code, which they used to complete their surveys and was used to keep their de-identified study data separately from information containing identifying information both in paper and electronic forms. Study data, identified by numerical code only, was shared with the National Database for Autism Research (NDAR) for those parents who gave explicit permission.

There was no independent data monitoring committee or personnel for this RCT as it was considered minimal risk (i.e., participants were completing surveys as primary research activity) and not required by the funding agency. The planned system for handling adverse event involved immediate reporting to the IRB for guidance and management; no adverse events occurred during the Pre-SRT period.

#### Statistical analyses and power

The overall study design has multiple levels of outcome measurements, collected at multiple time points. Parents and toddlers are nested within different PCP practices or EI programs, providers are nested within programs/practices, and both parents/children and providers are nested within different communities. We will use Generalized Linear Mixed Models (GLMMs) as the primary data analytic strategy to address the study research aims because this model type: (1) accounts for the nested structure, or multiple levels, of the data collected; (2) can be used to model change over time (i.e., both intercept and slope); and (3) can handle missing data for a specific survey without deletion of entire records [37, 38]. Because we expect that multiple providers nested within the same organization may have similar practices (due to clinical practice guidelines in place within different healthcare systems), we will use intraclass correlation coefficients (ICCs) to describe the extent to which providers’ responses are correlated within settings for the outcomes examined at the provider and caregiver/child level. Higher ICCs will reduce our power to detect change over time.

For child outcomes*,* the planned sample size would allow the detection of small to moderate differences between the Pre- and Post-SRT cohorts at 80% power. For child social communication, for example, the sample size would allow to us to detect a minimum increase of 40% more for the Post-SRT cohort relative to the Pre-SRT cohort assuming a similar amount of variation for both groups. For provider outcomes*,* based on inclusion of an estimated 40 PCP providers, we would have sufficient sample size to detect approximately 22% point differences between Pre- and Post-SRT intervention reports at 80% power, assuming that 50% of PCPs are implementing universal screening. With a sample of 80 EI providers, we would have sufficient sample size to detect minimum 15% point differences between Pre- and Post-SRT intervention reports at 80% power.

## Discussion

This paper describes a multi-system pragmatic trial of the Screen-Refer-Treat (SRT) service delivery model, a first of its kind, designed to promote an integrated and coordinated approach for early detection and specialized intervention for toddlers suspected of having ASD. Initial process data from the pre-intervention (Pre-SRT) phase highlight some of our successes, challenges, and “lessons learned” with respect to primary study activities involving provider and family recruitment and data collection, which are outlined below.

### Recruitment and retention of providers

We were successful in meeting–and exceeding–our enrollment targets for providers (58 PCPs and 87 EI providers; see Table 4). The number of PCP practices per county ranges from 1 to 4, and the number of participating EI program ranges from 1 to 5. However, the process of obtaining provider buy-in and organizational approvals took considerably longer than estimated. During the recruitment phase, the research team made multiple trips (1–3 per site) to meet with the PCP practices and EI agencies; these trips often required up to 2 months advance scheduling. Furthermore, some of the PCP practices required their own approval processes before enrolling providers. The turnaround time for these local approvals required another 2–5 months, significantly extending the start-up phase and delaying the start of data collection by about a year.

**Table 4:** Demographic Characteristics of PCPs and EI Providers

	PCPs (*n* = 58)	EI Providers (*n* = 87)
County: # (%)		
Spokane	9 (15.5)	60 (68.9)
Yakima	19 (32.8)	10 (11.5)
Skagit	16 (27.6)	6 (7.0)
Lewis	14 (24.1)	11 (12.6)
Gender: # (%)		
Female	41 (70.7)	77 (88.6)
Male	14 (24.1)	5 (5.7)
No response	3 (5.2)	5 (5.7)
Race: # (%)		
White	43 (74.1)	79 (90.8)
Other	12 (20.7)	5 (5.7)
No response	3 (5.2)	3 (3.5)
Ethnicity: # (%)		
Hispanic	1 (1.7)	3 (3.4)
Non-Hispanic	47 (81.1)	71 (81.7)
No response	10(17.2)	13 (14.9)
Professional Background: #(%)		
Medical doctor	42 (72.4)	N/A
Nurse Practitioner/ Physician Assistant	16 (27.6)	N/A
Speech-Language Pathologist	N/A	38 (43.7)
Occupational Therapist	N/A	20 (23.0)
Physical Therapist	N/A	11 (12.6)
Family Resource Coordinator	N/A	5 (5.8)
Other	N/A	13 (14.9)
No response	N/A	0 (0)
Years of Experience: M (SD)	13.72 (9.56)	15.20 (11.32)

Retention of providers has been moderate-to-high (75% for PCPs and 85% for EI providers) over the first 22 months. To promote retention, research team leaders made quarterly visits to each practice and program. This has been challenging for the more distant counties and those with several practices, as scheduling multiple visits on the same day was rarely possible. Other strategies employed to sustain our presence in communities included: having lunch delivered to PCP practices and EI programs; sending providers “SRT Network”-branded posters and mugs; having “check-in” phone calls; and offering incentives for recruitment efforts and for completing surveys. Additionally, for the two counties farthest from Seattle, community liaisons were hired to facilitate ongoing communication between enrolled providers and the research team.

Overall, the choice of a pragmatic trial and stepped-wedge cluster design worked to our advantage for engagement and retention because providers: (1) were active participants in determining procedures for implementing the SRT model, enabling them to optimize efficiency and fit in their sites; and (2) were assured of receiving training and access to new tools. The high rates of retention even in the remote sites likely reflects both our personalized attention and the presence of locally-based liaisons.

### Recruitment and retention of pre-SRT families

We experienced significant challenges in contacting families who signed “permission to contact” forms, particularly those recruited from PCP practices, and did not meet our planned enrollment targets for the ASD Concerns group. Of the 894 referrals from PCPs, 260 (29%) enrolled in the study and 243 (27%) completed surveys. 484 (54%) did not respond to our numerous attempts at contact, and of the 410 families we reached via telephone, 22 (5.4%) were ineligible. Recruitment of families from EI programs exhibited the reverse pattern, in that fewer families were referred (*n* = 151), but a larger proportion of those referred were enrolled (*n* = 85; 56%) and completed surveys (74; 49%). Among the referred families, 28 (19%) did not respond to our attempts at contact, and 14 of those contacted (11%) were ineligible. Altogether, of the 317 families who enrolled and completed surveys, 65 (21%) were in the ASD Concerns group, 68 (21%) were in the DD Concerns group, and 184 (58%) were in the No Concerns group (see Table 5). Our enrollment of 43 Hispanic families, 14% of all whom enrolled and submitted data, was lower than the 20% we anticipated based on census data.

**Table 5:** Demographic Characteristics of Enrolled Families

	ASD Concerns (*n* = 65)	DD Concerns (*n* = 68)	No Concerns (*n* = 184)
Toddler age (in months) at entry *M* (*SD*)	27.63 (5.53)	23.28 (4.87)	20.56 (1.32)
Caregiver age (in years) at entry *M* (*SD*)	33.42 (8.03)	34.16 (5.23)	32.13 (5.09)
Toddler sex # (%)			
Female	23 (35.4)	25 (36.8)	98 (53.3)
Male	42 (64.6)	43 (63.2)	86 (46.7)
No response	0 (0)	0 (0)	0 (0)
Parent race # (%)			
White	55 (84.6)	57 (83.8)	169 (91.8)
Other	9 (13.9)	9 (13.3)	12 (6.6)
No response	1 (1.5)	2 (2.9)	3 (1.6)
Parent ethnicity # (%)			
Hispanic	12 (18.5)	11 (16.2)	20 (10.9)
Non-Hispanic	53 (81.5)	56 (82.3)	163 (88.6)
No response	0 (0)	1 (1.5)	1 (0.5)
Parent Highest level of education attained #(%)			
No college degree	38 (58.4)	26 (38.3)	73 (39.7)
College degree (2-year or Bachelor’s degree)	23 (35.4)	28 (41.2)	82 (44.6)
Graduate degree	4 (6.2)	12 (17.6)	29 (15.7)
No response	0 (0)	2 (2.9)	0 (0)

Some of the challenges we experienced with family enrollment are consistent with reports indicating that families who live outside of metropolitan areas are underrepresented in mental health services research [39, 40]. Known impediments to non-participation, include low population density from which to recruit, lack of familiarity with research as a cultural practice [41, 42], and inadequate research infrastructure to support research participation. Studies that target remote populations might benefit by establishing decentralized research infrastructures to provide a sustained research presence for recruiting and retaining participants [43, 44]. Trusted local staff could function to personally recruit eligible families and consult to PCP and EI staff as they implement intervention and study procedures.

Additional barriers affect research participation for Hispanic families. We found that having Spanish language recruitment materials and surveys was insufficient to meet recruitment goals for this group. Our community liaisons reported that many Hispanic families work as farm laborers, and cited lack of telephone availability during daytime hours and outdated contact information due to frequent moves as potential challenges to research participation. In addition, recent changes in federal immigration policy may have impeded research participation. Future studies may benefit from hiring full-time research team staff who live locally and can dedicate more time building trust and awareness about the research, and potentially conduct in-person follow-up with families regarding study eligibility screening, consenting, and completing surveys.

Additionally, while our original plan was to recruit two groups of families (i.e., those with ASD Concerns and those with No Concerns), it became apparent that both PCPs and EI providers were hesitant to talk to parents about a study that referred to “ASD concerns.” Even when EI providers had ASD concerns about children, they were reluctant to share their concerns with families until it was time for the child to transition out of EI services into school services. Several adaptations were made in an effort to increase recruitment of the ASD Concerns group. First, the criteria for study eligibility were broadened to include a third group of toddlers for whom there were concerns about more general developmental delays. Second, we provided sample scripts and conducted in-person coaching with EI providers about how to introduce the study to parents in an encouraging manner. Third, we extended the recruitment period beyond the initial 6-month window. Although these measures were somewhat effective, our recruitment numbers for the ASD Concerns group remain lower than projected. With only a quarter of sample size as we initially planned, the power to detect our projected effect sizes on many outcomes will be seriously reduced. Still, we plan to carry out all the planned analyses. But the inference will be interpreted with caution. Instead of treating the analysis as confirmatory, we will focus on the direction and magnitude of the effect sizes, which will serve as effect size estimates for future, well powered, confirmatory studies. In addition, the current sample size should still provide enough power (> 90%) to detect an absolute increase of 30% in level-2 screening in EI Pre- vs. Post-SRT periods.

### Data collection

#### Provider and parent surveys

Of the 58 enrolled PCPs, 44 (76%) completed their first two Practices Surveys. Of the 87 enrolled EI providers, 76 (87%) completed their first two Practices Surveys. Of the 317 parents enrolled who contributed data, 275 (87%) completed surveys at two or more time points.

#### Patient-level services data

We encountered significant obstacles to the completion of the PCP Checklist and EI Checklists, which were developed to collect information on the services delivered to individual children. While we anticipated that 1120 PCP checklists would be completed by PCPs during the first 6 months of data collection, that milestone was not met until 12 months into the study. Feedback revealed that the PCP Checklist procedure was not well integrated into their office work flow; as a result, strategies for modifying their work flow were developed through consultation with the office manager and PCPs. In addition, checklist completion goals were established for each practice, and incentivized with: (1) their choice of materials for their practice (e.g., books or toys for their waiting area), and (2) performance feedback in the form of run charts, enabling them to compare their checklist completion rates with other (anonymized) practices. We also met with challenges obtaining EI Checklists, as it was difficult for EI programs to identify staff members who had the time and knowledge to provide the requested information. Of the 85 families enrolled in EI, only 54 checklists (64%) were completed. In addition, considerable delays in returning the EI Checklists rendered it difficult to compare parent and provider reports of services delivered.

## Conclusion

Provider hesitancy to discuss ASD concerns, time constraints, and challenges in modifying work flow, which are considered significant contributors to the “theory to practice/policy” gap [45], also emerged as the primary implementation barriers for *research activities*. Hesitancy to mention ASD concerns was one of the primary reasons that family recruitment rates were lower than expected. Unlike trials that focus on treatment for *diagnosed* populations/samples, the PCPs and EI providers in the current study had the added challenge of recruiting families whose children did not have an ASD diagnosis, and who may not have been aware of ASD as a potential concern for 18-month-olds. While modifications to recruitment eligibility and materials were made to remove ASD as the focal point, providers still experienced difficulty in presenting recruitment materials to families. Overall, these challenges parallel those documented in other preventive intervention studies [46–48], including other ongoing projects within the NIMH ASD PEDS network, which are conducting research on screening tools and intervention for children at risk for ASD [49]. Applying strategies from Quality Improvement [50] and Implementation Facilitation [51] approaches (which aim to reduce the “gap” between best practices and community implementation) at the study outset may have prevented some of these implementation barriers from arising.

In sum, the development of the SRT model was inspired by the need for an earlier and more continuous route to specialized services for families of toddlers with ASD or suspected ASD, and we chose a preventive intervention framework to meet that need. We have learned many lessons about study implementation during the pre-intervention phase of trial that may be helpful to others designing future pragmatic trials. For example, allowing for an extended start-up phase of 6–12 months may be needed to obtain provider buy-in, IRB approvals from multiple institutions, input and feedback from providers and key stakeholders, and adjustment of clinic workflow procedures to implement key study procedures. However, this may be challenging within the context of traditional funding mechanisms. In contrast, the alternative of conducting smaller feasibility/pilot studies [52] is unlikely to reveal the multitude of issues and complexities that arise within a larger scale study, especially one that targets multiple, interacting service delivery systems. Other ASD researchers are urged to consider the use of pragmatic trials to bridge the gap between lab-based advances and community practices. Although the ultimate success of the SRT model is yet to be determined, the results of this study will provide key insights about how to best introduce/disseminate it given that: (1) a network of information and engagement has been established with stakeholders (e.g., Department of Health) and existing infrastructure (i.e., Part C EI); and (2) the model has high ecological validity since it is being evaluated in a “usual care” setting (i.e., community), rather than a carefully controlled clinical lab setting, which likely reduces barriers to future transportability.

## Data Availability

The PI and Co-Investigators will have access to the final dataset and a portion of the data will be available on the National Database for Autism Research for researchers that apply through NIMH. Potential authors for future publications will be expected to adhere to American Psychological Association guidelines.

## Abbreviations

AAP: American Academy of Pediatrics
ASD: Autism Spectrum Disorder
CEU: Continuing education unit
CME: Continuing medical education
DD: Developmental delays
EI: Early intervention
GLMMs: Generalized Linear Mixed Models
HIPAA: Health Insurance Portability and Accountability Act
ICCs: Intraclass correlation coefficients
IDEA: Individuals with Disabilities Education Act
IRB: Institutional Review Board
M-CHAT-R/F: Modified Checklist for Autism in Toddlers-Revised, with Follow Up
MOC: Maintenance of certification
NDAR: National Database for Autism Research
NIMH ASD PEDS: National Institute of Mental Health Autism Spectrum Disorder Pediatric, Early Detection, Engagement and Services
PCP: Primary care providers
PI: Principal investigator
RCT: Randomized controlled trial
REDCap: Research electronic data capture
RIT: Reciprocal Imitation Training
SRT: Screen-Refer-Treat
STAT: Screening Tool for Autism in Toddlers
TA: Technical assistance
UW: University of Washington
WA: Washington
WHO Quality Of Life-BREF: World Health Organization Quality of Life abbreviated version
WIC: Women, Infants, and Children
